# Anticancer and antibacterial potentials induced post short-term exposure to electromagnetic field and silver nanoparticles and related pathological and genetic alterations: in vitro study

**DOI:** 10.1186/s13027-022-00416-4

**Published:** 2022-02-04

**Authors:** Aly Fahmy Mohamed, Mohamed Nasr, Mohamed E. Amer, Tamer M. M. Abuamara, Wagih M. Abd-Elhay, Hassan Fathy Kaabo, Emad Eldin R. Matar, Laila E. El Moselhy, Tamer Albasyoni Gomah, Mohammed Abd EL-Fatah Deban, Rania Ibrahim Shebl

**Affiliations:** 1 International Center for Training and Advanced Researches (ICTAR-Egypt), Cairo, Egypt; 2 Histology Department, Faculty of Medicine, Al-Azhar University, Cairo, Egypt; 3 Histology Department, Faculty of Medicine, Al-Azhar University, Damietta, Egypt; 4 Pathology Department, Faculty of Medicine, Al-Azhar University, Cairo, Egypt; 5 Microbiology and Immunology Department, Faculty of Pharmacy, Ahram Canadian University (ACU), 4th Industrial Zone, Banks Complex, 6th October City, Cairo, Egypt

**Keywords:** Antibacterial, Anticancer, Electromagnetic field, Silver nanoparticles, Cell cycle, Apoptosis, Oxidative stress

## Abstract

**Background:**

Resistance to antibiotics and anticancer therapy is a serious global health threat particularly in immunosuppressed cancer patients. Current study aimed to estimate the antibacterial and anticancer potentials of short-term exposure to extremely low frequency electromagnetic field (ELF-EMF) and silver nanoparticles (AgNPs) either in sole or combined form.

**Methods:**

Antibacterial activity was evaluated via determination of the bacterial viable count reduction percentage following exposure, whereas their ability to induce apoptosis in breast cancer (MCF-7) cell line was detected using annexin V-fluorescein isothiocyanate and cell cycle analysis. Also, oxidative stress potential and molecular profile were investigated.

**Results:**

ELF-EMF and AgNPs significantly (*p* < 0.01) reduced *K. pneumonia* viable count of compared to that of *S. aureus* in a time dependent manner till reaching 100% inhibition when ELF-EMF was applied in combination to 10 µM/ml AgNPs for 2 h. Apoptosis induction was obvious following exposure to either ELF-EMF or AgNPs, however their apoptotic potential was intensified when applied in combination recording significantly (*p* < 0.001) induced apoptosis as indicated by elevated level of MCF-7 cells in the Pre G1 phase compared to control. S phase arrest and accumulation of cells in G2/M phase was observed following exposure to AgNPs and EMF, respectively. Up-regulation in the expression level of p53, iNOS and NF-kB genes as well as down-regulation of Bcl-2 and miRNA-125b genes were detected post treatment.

**Conclusions:**

The antibacterial and anticancer potentials of these agents might be related to their ability to induce oxidative stress, suggesting their potentials as novel candidates for controlling infections and triggering cancer cells towards self-destruction.

## Introduction

The progressing emergence and the rapid spread of bacterial resistance to antibiotics is considered an alarming world-wide health problem which strengthens the need for alternative therapies. This continually growing problem of antibiotic resistance not only endangers the public health, but also endures a massive negative impact on the economic development due to delayed hospitalization and recovery time in addition to the need for expensive medications as well as specialized care for patients [1]. Many researchers have directed their efforts to manage the problem of antibiotic resistance via estimating the effectiveness of new antibacterial agents either alone or in combinations [2]. In the same context, breast cancer is considered the second common leading cause of cancer death among women [3]. Although many cases of cancer initially respond to chemotherapy, but resistance is usually developed later [4] in addition to the undesirable side effects that are associated with the currently available chemotherapeutic drugs. Thus, there is also an urgent demand for developing biocompatible and cost-effective anticancer agents [5].

Extremely low frequency electromagnetic field (ELF-EMF) is one of the most recent applications that exhibited significant interactions with the living cells. However, the mechanism of this interaction is still not clarified. Recent studies were carried out to assess the biological influence of such fields on different types of living cells especially on bacterial cells. Multi-directional alterations following bacterial exposure to ELF-EMF were reported such as ultra-structural and growth kinetics changes [6]. Whereas, other studies found that ELF-EMF could enhance or suppress bacterial functional parameters. Therefore, investigating the influence of ELF-EMF on bacteria is essential not only for evaluating the impact of environmental stress on biological systems, but also to explore the possibility of using the ELF-EMF to control the resistance to antibiotics [7].

ELF-EMF generating medical devices were also applied for treatment of cancer patients in intensive care units. Consequently, during the outgoing 25 years researchers tried to investigate the impact of exposure to ELF-EMF on cellular and molecular behavior in addition to its effect on cancer cell metabolism. It was found that exposure to 50/60 Hz magnetic field promoted changes in signal transduction pathways that were directly associated to proliferative processes [8]. A study reported that electromagnetic field (EMF) could selectively hinder the oxidation–reduction signaling in cancer cells relying on the differential electrical behavior between the cancer and the normal cells. This signaling plays a key role in blocking cellular functions leading to induction of programmed cell death [9]. Recently, a study reported that the tumor suppressive effects of the ELF‐EMF could present a new approach for the treatment of breast cancer if this technology is clinically applied [10]. It was demonstrated that the ability of pulsed low-frequency EMF to modify the membrane integrity of cancer cells presents a new strategy in anticancer therapy. These pulsed magnetic fields (PMF) could selectively destruct the cancer cell membranes without the use of ionizing radiation or cytotoxic agents. Thus, these fields could be applied as adjuvants in cancer therapy to facilitate the delivery of anticancer agents to tumor cells [11]. It was also found that EMF enhanced the in vivo anti-tumor efficacy of cisplatin against Lewis lung carcinoma cells [12]. Moreover, the exposure of glioblastoma brain cancer cells to a combination between EMF and the anticancer agent temozolomide enhanced the apoptosis via elevated expression of P53, Bax, and Caspase-3 (pro-apoptotic) genes while decreasing the expression levels of Bcl-2 and Cyclin-D1 (anti-apoptotic) genes [13].

Additionally, the recent advances in nanotechnology offered new horizons in nanomedicine, facilitating the synthesis of nanoparticles (NPs) that could be applied as powerful weapon against pathogenic bacteria and cancer cells [14].

Among nanotechnology-based therapeutics, AgNPs attracted the attention of many researchers due to their distinctive characteristics and marked therapeutic potential in treating different diseases [14]. The antimicrobial activities of AgNPs either alone [15] or in composites with polymer [16] have been demonstrated in addition to their anticancer [17] as well as their antiangiogenic potentials [18]. AgNPs are now considered a valuable and non-traditional alternative to antibiotics with high antimicrobial potential against multidrug-resistant (MDR) Gram-positive and Gram-negative bacterial pathogens [19]. It was also reported that AgNPs inhibited the proliferation of human glioblastoma cells [20] as well as human breast cancer (MCF-7) cells [21]. It was also found that AgNPs stimulated pro-apoptotic genes leading to interference with normal cellular functions and induction of programmed cell death. A study reported that AgNPs induced apoptosis in NIH3T3 fibroblast cells is mediated via generation of reactive oxygen species (ROS) and activation of Jun N-terminal kinases (JNK) pathway leading to mitochondrial dependent apoptosis [22]. Recently, the microenvironmental influence of titanium dioxide NPs as a mechanical stimulus on cancer cells has been also observed [23]. In addition to the reported concentration dependent metabolic disturbing effect of graphene oxide nanosheets on MCF-7 cells [24]. This alteration in the metabolomic profiling of cancer cells could control many malignant properties that are responsible for tumorigenesis [25]. It was also found that the mechanobiological studies of AgNPs in cancer metabolomics suggested that AgNPs might be promising tools that could be explored to develop enhanced anticancer therapy [26].

Consequently, the present study aimed to evaluate the antibacterial and the anticancer potentials of short-term exposure to ELF-EMF and AgNPs either in sole or combined form at different time intervals. The mechanism of action of these agents were elucidated via estimating their ability to induce oxidative stress and their effects on the antioxidant enzymes. Apoptosis induction ability in MCF-7 cells was examined using different staining techniques as well as cell cycle analysis. Monitoring the expression profiles of five genes namely p53, inducible nitric oxide synthase (iNOS) and nuclear factor-kappaB (NF-kB), B cell lymphoma-2 (Bcl-2) and microRNA-125b (miR-125b) were also investigated following treatment.

## Materials and methods

### Bacterial strains and culture conditions

Clinical isolate of *Staphylococcus aureus* (*S. aureus*) and *Klebsiella pneumonia* (*K. pneumonia*) was used as a model for Gram-positive and Gram-negative bacteria, respectively. Both isolates were obtained from Kasr Al-Ainy Teaching Hospital. *S. aureus* was isolated from pus specimen and identified using Gram stain, biochemical catalase and coagulase tests in addition to formation of golden yellow colonies on nutrient agar as well as mannitol fermentation on mannitol salt agar. *K. pneumonia* was recovered from urine specimen and the isolate was cultured on blood agar and MacConkey’s agar. Colonies were identified as *Klebsiella pneumonia* by biochemical reaction and confirmation was carried out using the API-20E (BioMérieux-France) test system according to the manufacturer’s instructions.

The bacterial concentration of each isolate was adjusted to an optical density (OD) of 0.1 at 600 nm which is equivalent to 10^8^ colony forming units/ml (CFU/ml) and inoculated on nutrient agar plate (pH of 7.0 ± 0.2) followed by incubation for 24 h at 37 °C. Before performing each experiment, three colonies from each isolate  were collected from each agar plate and inoculated in 5 mL nutrient broth to obtain fresh subcultures.

### Cell culture

Breast cancer (MCF-7) cells (HTB-22) were kindly supplied from the International Center for Training and Advanced Researches (ICTAR-Egypt). Cells were cultured in RPMI-1640 media (Hyclone-USA), supplemented with 10% heat inactivated fetal bovine serum (FBS), 100 U/ml penicillin and 100 μg/ml streptomycin. Cells were incubated in a humidified atmosphere of 5% CO_2_ at 37 °C (Jouan-France) till reaching confluency.

### Characterization of commercial AgNPs

AgNPs were purchased as a commercial product by Nawah scientific (Cairo-Egypt). Characterization of the particle size distribution and surface charge of the prepared particles were determined using Zetasizer Nano-ZS (Malvern Instruments-UK). Morphology and mean size of AgNPs were  determined using Field Emission Scanning Electron Microscopy (FESEM) (JSM-7600F, Joel-Japan), at accelerating voltages of 15 kV as previously described by Alkawareek et al. [27].

### Antibacterial potential

Determination of minimal inhibitory concentration (MIC) of the tested AgNPs against *S. aureus* and *K. pnemonia* was carried out using broth micro dilution method according to the CLSI reference standards [28]. One hundred microliter of Muller-Hinton broth (MHB) (Oxoid-UK) were distributed in 96 multi-well microtiter plates (TPP-Swiss). Double fold serial dilutions of AgNPs were performed. Bacterial inoculum was prepared by adjusting the OD at 600 nm of the previously prepared bacterial suspension to reach a turbidity of 0.5 McFarland standard which is equivalent to 10^8^ CFU/ml. The prepared suspension was further diluted and inoculated in all plates at a final concentration of 5 × 10^5^ CFU/ml. Positive and negative control in each plate were considered. Plates were incubated at 37 °C for 24 h and examined visually against dark background for absence or presence of turbidity. The MIC was determined as the lowest concentration of AgNPs with no visible bacterial growth compared to control. Twenty µl from each well with no observed bacterial growth were further inoculated on the surface of  agar plates and incubated overnight at 37 °C. The lowest concentration of AgNPs that kills > 99.9% of the initial bacterial inoculum is considered as the minimum bactericidal concentration (MBC).

### Electromagnetic field treatment

Fresh subcultures of *S. aureus* and *K. pneumonia* at a final concentration of 5X10^5^ CFU/ml were aliquoted in sterile polystyrene plastic screw capped tubes and treated with different concentrations of AgNPs (2.5, 5 & 10 µM/ml) at 37 °C for 1 & 2 h interval. In the meantime, another set of tubes containing the same concentrations of bacteria and AgNPs were exposed to ELF-EMF of 1 m Tesla for the same time interval at 37 °C. Whereas, other tubes inoculated with the same concentration of bacteria were treated with ELF-EMF in absence of AgNPs. AgNPs or ELF-EMF untreated bacterial suspensions were considered as positive  control. At the end of the treatment period, samples were obtained from all tubes to determine the bacterial viable count. The percentage reduction in the viable count was calculated for all examined samples compared to control.

MCF-7 cells pre-cultured 75 cm^2^ tissue culture flasks (SPL-Korea) were dissociated using 0.25% trypsin–EDTA (Lonza-Swiss) post decanting the exhausted growth media. Detached cells were cold centrifuged (Jouan, Ki-22-France), phosphate buffer saline (PBS) washed and resuspended in 20 ml RPMI-1640 serum free media. Cells were equally aliquoted in sterile polystyrene tubes, treated with AgNPs IC_50_ value and exposed to ELF-EMF of 1 m Tesla for 1 and 2 h interval. Similar concentrations of MCF-7 cells were treated with ELF-EMF at the same conditions but in absence of AgNPs. Untreated control cells were also considered. ELF-EMF treated cells either in presence or absence of AgNPs were examined for pathological changes in addition to cell cycle and molecular analysis as well as biochemical tests.

### Cytotoxicity

Cytotoxic effect of different concentrations of AgNPs was determined using 3-(4,5-dimethylthiazol-2-yl)-2,5-diphenyl-tetrazolium bromide (MTT) assay, where growth media were  decanted from 96-well micro titer plates pre-cultured with MCF-7 cells. AgNPs were applied in double fold serial dilutions to MCF-7 precultured plates. Untreated wells served as negative control and plates were incubated at 37 °C for 24 h. Post incubation, the plates were washed three times with PBS as 250 μl/well. Fifty μl of MTT solution (0.5 mg/ml) were added to each well and plates were incubated for further 4 h at 37 °C. Plates were PBS washed three times and the formed blue colored formazan was dissolved using 50 μl/well DMSO (Sigma Aldrich-USA) followed by shaking the plates for 10 min at room temperature. Optical density (OD) was measured at 570 nm using ELISA plate reader (Biotek, ELX-800-USA). The percentage of cellular viability was calculated, and the half maximal inhibitory concentration (IC_50_) was determined as the concentration resulting in 50% inhibition of cellular growth following 24 h exposure to AgNPs compared to the untreated control cells using GraphPad prism software version 5 (S. Diego-USA) [29].

### Hematoxylin and eosin staining

Fifty micro liters of ELF-EMF treated MCF-7 cells either in presence or in absence of AgNPs were dispensed on clean slides (3 slides for each treatment). Slides were air-dried, methanol fixed and rehydrated using descending concentrations of alcohol (100%, 90%, 75% and 50%). Slides were washed with distilled water for 5 min. The slides were immersed in filtered hematoxylin stain for 3 min and washed with distilled water twice followed by immersion in filtered eosin stain for 5 s and washed with distilled water. Dried slides were immersed in xylene followed by mounting with Canada balsam. The coverslips were mounted to each slide and left to air dry. Microscopic fields (100X) were photographed using digital camera (Canon-Japan), connected to a light microscope. The photomicrographs were evaluated for the presence of morphological features of apoptosis [30].

### Apoptosis detection

ELF-EMF treated MCF-7 cell suspension, mixed with and without AgNPs were quantitatively examined for detection of apoptosis using annexin V-fluorescein isothiocyanate (FITC) apoptosis detection kit (Trevigen-USA). Briefly, treated and untreated cells were PBS washed and collected by centrifugation. Cells were dark incubated for 15 min at room temperature with 100 μl annexin-V incubation reagent: 10 µl (10 X) binding buffer 1, 10 µl propidium iodide (PI), 1 µl annexin V-FITC and 79 µl de-ionized water. Samples were treated with 400 μl binding buffer (1 X) and analyzed using flow cytometer within 1 h for maximal signal.

### Cell cycle analysis

ELF-EMF treated MCF-7 cell suspension, mixed with and without AgNPs were processed for cell cycle analysis. Cell cycle distribution was examined by measuring the DNA content of nuclei labeled with propidium iodide (PI). Cell suspensions were collected by cold centrifugation, washed with 1 ml cold PBS, centrifuged, and fixed in 70% cold ethanol (Sigma Aldrich-USA) at + 4 °C for 24 h. Cells were re-suspended in PBS containing 40 μg/ml PI, 0.1 mg/ml RNase and 0.1% (v/v) Triton X-100. Post dark incubation at 37 °C for 30 min, the cells were analyzed using flow cytometer (Becton–Dickinson, San Jose, CA, USA) equipped with an argon ion laser at a wavelength of 488 nm. The cell cycle and sub-G1 group were determined and analyzed.

### Real time-PCR

Total RNA was extracted from control, 1 and 2 h ELF-EMF treated MCF-7 cells in presence and in absence of AgNPs using RNeasy Mini Kit (Qiagen-USA) according to manufacturer’s instructions. The concentration and purity of the extracted RNA was evaluated using Beckman dual spectrophotometer (Beckman-USA). The expression level of apoptosis-related genes; P53 (F: 5’-TCA GAT CCT AGC GTC GAG CCC-3’ & R: 5’-GGG TGT GGA ATC AAC CCA CAG-3’), BCL-2 (F: 5’-GTG AAC TGG GGG AGG ATT GT-3’& R: 5’-GGA GAA ATC AAA CAG AGG CC-3’), NF-kB (F: 5'-CGC ATC CAG ACC AAC AAC A-3 & R: 5'-TGC CAG AGT TTC GGT TCA C-3'), iNOS (F: 5'-AGT ATG CAA TGA ATG GGG AA-3' & R: 5'-ATT CGA TAG CTT GAG GTA GA-3'), miR-125b (F: 5'-ACT GAT AAA TCC CTG AGA CCC TAA C-3' & R: 5'-TAT GGT TGT TCT GCT CTC TGT CAC-3')   and β-actin (F: 5'-AGA GCT ACG AGC TGC CTG AC-3' & R: 5'-AGC ACT GTG TTG GCG TAC AG-3') were determined using real-time PCR. Ten nanograms of the extracted total RNA from each sample were used for cDNA synthesis using high capacity cDNA reverse transcriptase kit (Thermo Fischer Scientific-USA). The obtained cDNA was subsequently amplified using Sybr Green I PCR master kit (Thermo Fisher Scientific Inc.- Lithuania) using StepOne apparatus (Applied Biosystems-Thermo Fischer Scientific), as follows: 10 min at 95 °C for enzyme activation followed by 40 cycles of 15 s at 95 °C, 20 s at 55 °C and 30 s at 72 °C for the amplification step. Changes in the expression of the target genes were normalized relative to the mean critical threshold (CT) values of β-actin as a housekeeping gene.

### Biochemical analysis

Assessment of reactive oxygen species, superoxide dismutase and catalase levels were determined in bacterial models as well as in MCF-7 cancer cell model following exposure to either ELF-EMF or AgNPs in addition to the combined treatment at different time interval. Bacterial models were treated with 5 µM/ml AgNPs, while MCF-7 cells were treated with IC_50_ of AgNPs.

### Reactive oxygen species (ROS)

Assessment of the generated reactive oxygen species was determined according to the manufacturer’s protocol using ELISA kit, Catalog No. K936-100.

### Superoxide dismutase (SOD)

SOD was evaluated using PromoKine kit-Catalog Number: PK-CA577-K335 (PromoCell-Germany) according to the manufacturer’s instruction. The assay is based on that superoxide dismutase is an antioxidative enzyme which catalyzes the dismutation of the superoxide anion into hydrogen peroxide and molecular oxygen. The rate of the reduction with a superoxide anion is inhibited by SOD. Therefore, activity of SOD could be determined via evaluation of the percentage inhibition rate colorimetrically.

### Catalase (CAT)

Catalase enzyme activity was evaluated using an Amplex® red catalase assay kit (Molecular Probes Inc.). The principle of the assay depends on that Amplex Red reagent reacts with H_2_O_2_ in the presence of horseradish peroxidase (HRP) and catalase enzyme to produce highly fluorescent resorufin. The assay was carried out according to the manufacturer’s instructions. Briefly, catalase containing samples as well as control samples were diluted in reaction buffer and transferred to 96-well microplate (25 μl/well). Hydrogen peroxide prepared as 40 μM H_2_O_2_ was added to each well (25 μl/well). The reaction mixture was incubated for 30 min at room temperature followed by adding 50 μl/well Amplex Red/HRP. The plate was incubated for 30 min at 37 °C protected from light. The fluorescence intensity was evaluated in all wells by reading the plate using an excitation range of 530–560 nm and emission at 590 nm. The activity of catalase enzyme was determined by subtracting the sample value from that of negative control.

### Statistical analysis

All experiments were performed in three independent tests. Data were presented as the mean ± standard deviation (SD) and analyzed using one-way analysis of variance (ANOVA) and Tukey post-hoc test. Statistical analysis was carried out using statistical package for social sciences (SPSS) software (version 25), IBM, USA. Results were considered statistically significant at probability < 0.05.

## Results

### Antibacterial potential

Recorded data revealed that AgNPs showed greater inhibitory potentials on *K. pneumonia* compared to that in case of *S. aureus*, where the recorded MICs were in the order of 3.125 and 12.5 µM/ml, respectively. It was also observed that the recorded MICs exerted bactericidal activity on more than 99.9% of the initial bacterial inoculum, so the recorded MICs were considered as MBCs.

**Fig. 1:**
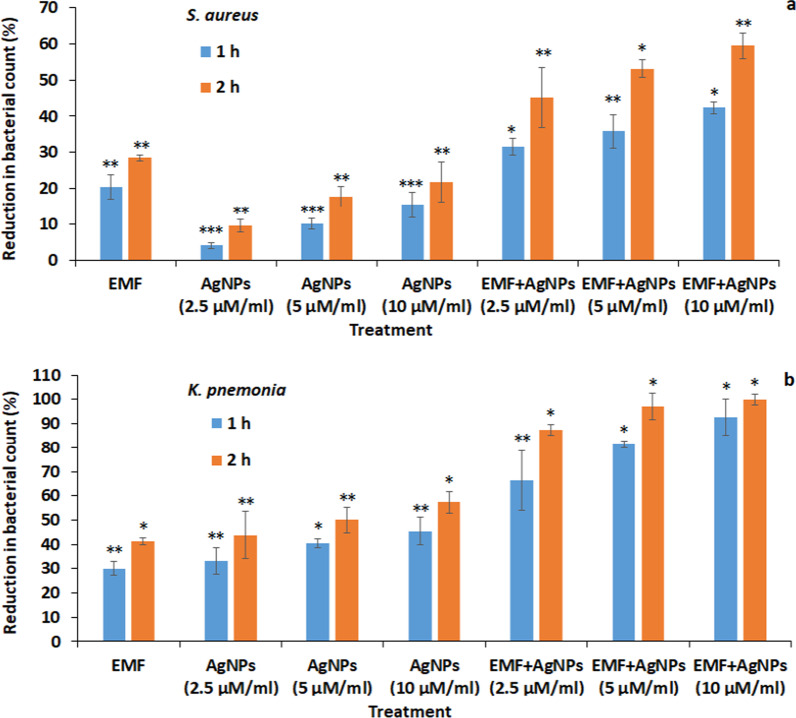
1a: Evaluation of antibacterial activity of sole EMF and AgNPs as well as EMF combined with AgNPs on *S. aureus* viable count at 1 and 2 h time interval. 1b: Assessment of the percentage reduction of *K. pneumonia* viable count post 1 and 2 h exposure to EMF, AgNPs and EMF in combination to AgNPs. *P < 0.001, **P < 0.01, ***P < 0.05

Results showed that *S. aureus* viable count reduction percentage post exposure to sole EMF for 1 and 2 h time interval was 20.4% ± 3.4 and 28.5% ± 0.9, respectively. At  the same time, AgNPs exhibited a statistically significant (*P* < 0.05) concentration dependent reduction in *S. aureus* recording 4.1% ± 0.8, 10.3% ± 1.5& 15.5% ± 3.5 post 1 h exposure to 2.5, 5 & 10 µM/ml AgNPs, respectively. Extension of the treatment period with AgNPs for 2 h resulted in higher reduction in the bacterial count by 9.6% ± 1.7, 17.7% ± 2.8 and 21.8% ± 5.6, respectively. An enhancement in the antibacterial activity was recorded when AgNPs were applied in combination to EMF, where the percentage reduction recorded 31.6% ± 2.2, 35.8% ± 4.6 & 42.3% ± 1.6 and 45.2% ± 8.3, 53.1% ± 2.5 and 59.4% ± 3.6 following 1 and 2 h treatment interval, respectively (Fig. 1a).

Similar pattern of antibacterial activity was observed in case of *K. pneumonia* but with statistically significant (*p* < 0.01) higher inhibitory potential compared to *S. aureus.* Treatment of *K. pneumonia* with EMF for 1 and 2 h resulted in reduction in  the bacterial count by 30.1% ± 2.9 and 41.3% ± 1.4, respectively. *K. pneumonia* viable count was reduced in a concentration dependent manner in the order of 33.2% ± 5.3, 40.5% ± 1.8& 45.4% ± 5.6 post 1 h exposure to 2.5, 5 and 10 µM/ml AgNPs, respectively. The inhibitory potentials were increased by extending the incubation period to 2 h recording 43.9% ± 9.7, 50% ± 5.4 and 57.3% ± 4.6, respectively. A higher reduction in the bacterial count was observed when AgNPs were applied in combination to EMF where they recorded 66.5% ± 12.3, 81.3% ± 1.5 and 92.6% ± 7.4 post 1 h treatment, respectively. In the same context, maximum inhibition was recorded following 2 h exposure to 2.5 and 5 µM/ml AgNPs in combination to EMF in the order of 87.4% ± 2.3 and 97.2% ± 5.5, respectively, till achieving 100% inhibition when EMF was applied to the bacterial suspension in presence of 10 µM/ml AgNPs (Fig. 1b).

### Biochemical analysis in bacterial models

**Fig. 2:**
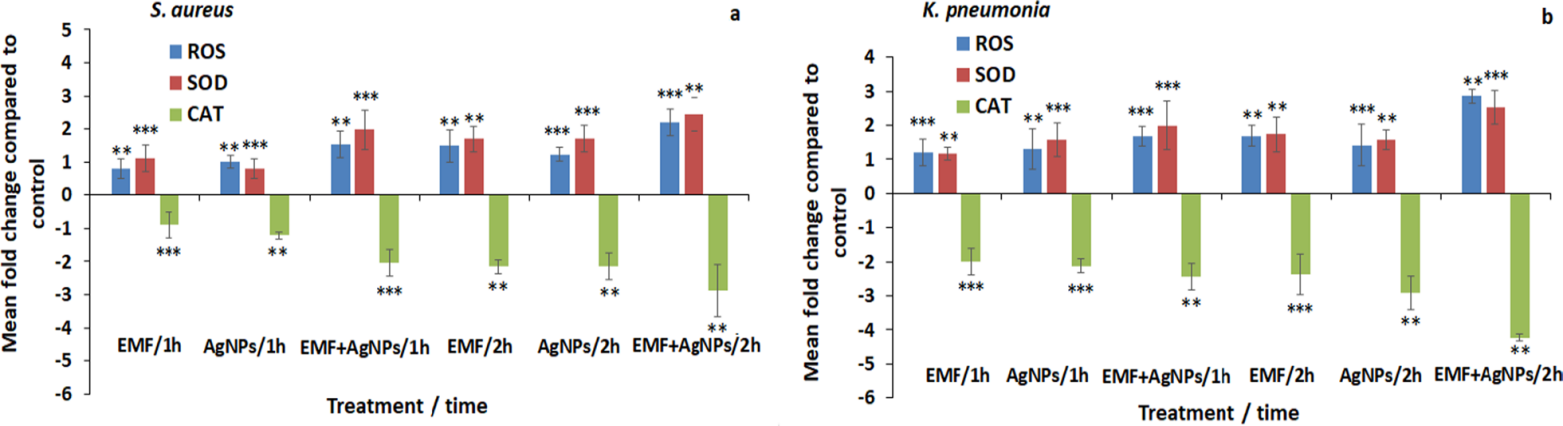
**a** Mean fold change in ROS, SOD and CAT in *S. aureus* 1 and 2 h post treatment with EMF, AgNPs and EMF + AgNPs compared to control. **b** Biochemical analysis of ROS, SOD and CAT levels following *K. pneumonia* exposure to EMF, AgNPs and EMF + AgNPs at 1 and 2 h time interval. **P < 0.01, ***P < 0.05

Evaluation of the ROS and SOD in *S. aureus* following 1 h exposure to EMF, AgNPs and EMF + AgNPs revealed elevated ROS levels by 0.8-, 1.0- and 1.52-fold as well as an increase in SOD by 1.12-, 0.8- and 1.98-fold compared to control, respectively. Further incubation for 2 h was accompanied by higher ROS and SOD levels in the order of 1.48-, 1.23- and 2.2-fold for ROS and 1.69-, 1.72- and 2.44-fold in case of SOD, respectively (Fig. 2a). *K. pneumonia* also showed elevated levels of ROS by a value of 1.2-, 1.3- and 1.68-fold as well as 1.69-, 1.42- and 2.86-fold 1 and 2 h following exposure, respectively. In addition to increased SOD levels by values of 1.17-, 1.58- and 2.0-fold as well as 1.73-, 1.58- and 2.52-fold 1 and 2 h post exposure, respectively (Fig. 2b).

On the contrary, treatment of *S. aureus* with EMF, AgNPs and EMF + AgNPs for 1 h showed a marked reduction in CAT levels by 0.9-, 1.21- and 2.04-fold as well as 2.16-, 2.14- and 2.88-fold reduction post 2 h exposure (Fig. 2a). Comparable reduced levels of CAT were detected in *K. pneumonia* post 1 h treatment recording 2-, 2.12- and 2.43-fold reduction as well as reduced CAT levels by 2.36-, 2.91- and 4.23-fold post 2 h treatment compared to control, respectively (Fig. 2b).

### Cytotoxicity

**Fig. 3:**
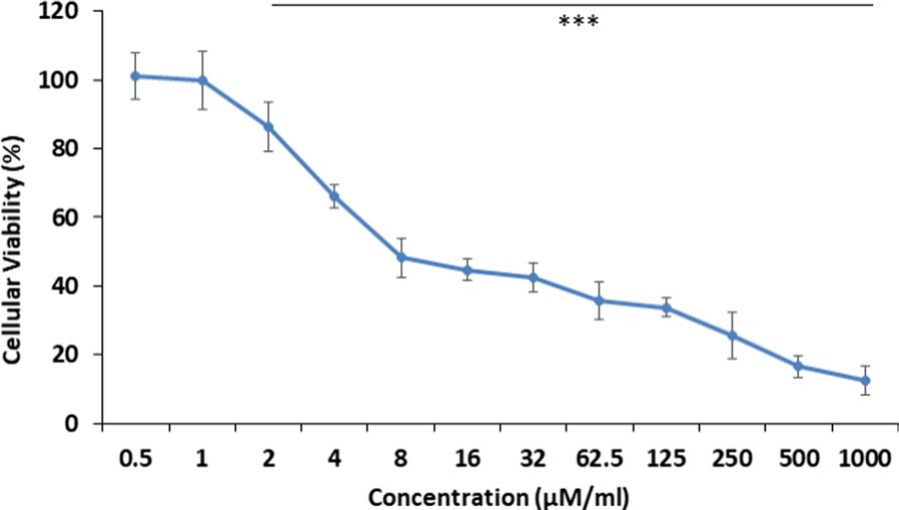
Evaluation of cellular viability of MCF-7 cells post 24 h treatment with AgNPs using MTT assay indicating elevated viability along with decreasing AgNPs concentration. ***P < 0.05

The cytotoxic effect of AgNPs 24 h post MCF-7 cells treatment was determined using MTT assay. Recorded data revealed that viability was concentration dependent, where the  viability increases as long as the concentrations of AgNPs decrease till reaching 100% viability at a concentration of 1 μM/ml. The calculated IC_**50**_ value of AgNPs was 4.15 ± 0.20 μM/ml (Fig. 3).

### Hematoxylin and eosin staining

**Fig. 4:**
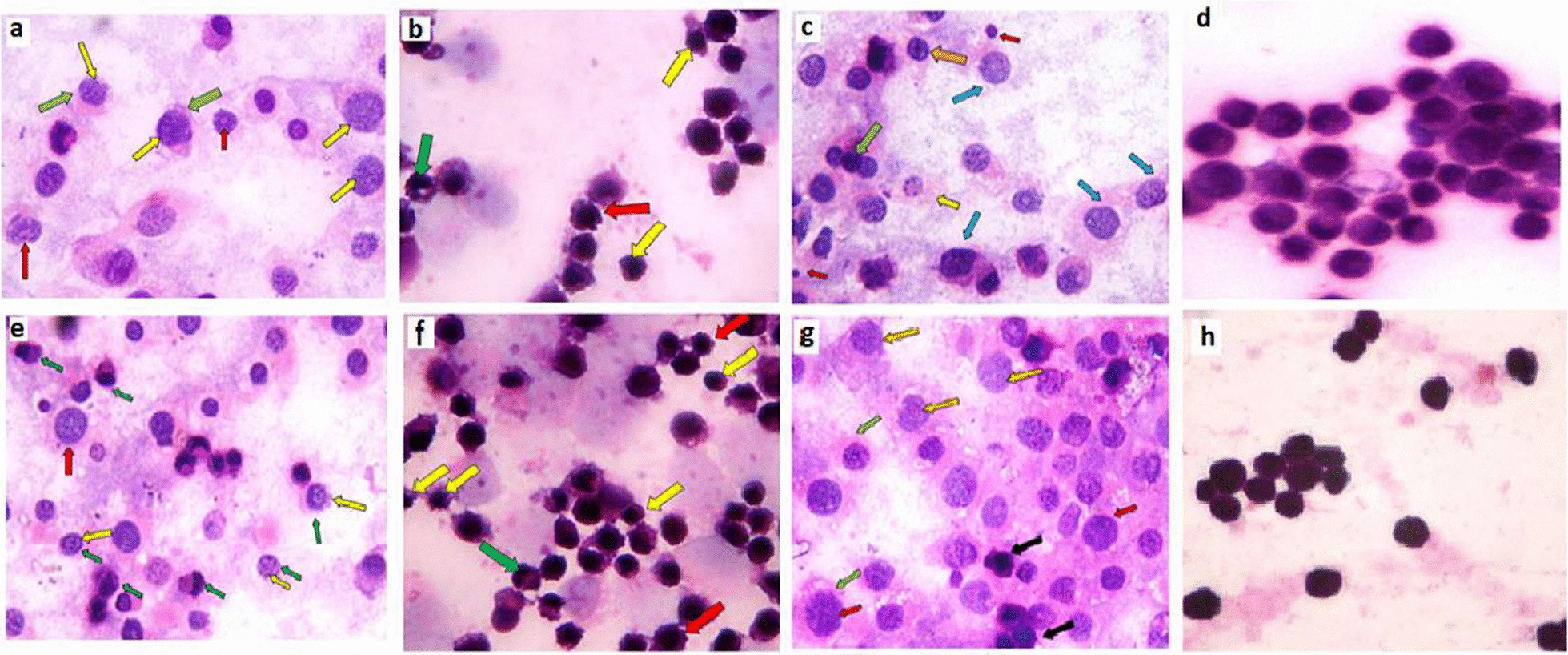
Pathological changes detected post MCF-7 cells exposure to sole ELF-EMF, AgNPs and ELF-EMF in combination to AgNPs at different time interval using hematoxylin and eosin staining. **a** MCF-7 cells photomicrograph post 1 h exposure to ELF-EMF showing swollen cells, swollen nuclei with mixed euchromatin and heterochromatin (Yellow arrows) as well as ruptured cell membranes (Green arrows) in addition to intranuclear eosinophilic structures (Red arrows). **e** Photomicrographs following 2 h treatment with ELF-EMF revealing the occurrence of apoptosis as indicated by the observed shrunken apoptotic cells with peripheral condensation of chromatin (Green arrows) as well as necrotic cells with mixed euchromatin and heterochromatin (Yellow arrows) and swollen cell with ruptured cell membrane (Red arrow). **b&f** Photomicrographs of MCF-7 cells post exposure to AgNPs for 1 and 2 h, respectively revealing the presence of shrunken apoptotic cells with shrunken nuclei (Yellow arrows), peripheral condensation of chromatin (Green arrows) and irregular cell membranes (Red arrows). **c** Swollen necrotic MCF-7 cells and swollen nuclei with mixed euchromatin and heterochromatin and ruptured cell membranes (Blue arrows) were detected post 1 h exposure to ELF-EMF in combination to AgNPs. Shrunken apoptotic cell (Green arrow) with peripheral condensation of chromatin (Orange arrow) in addition to secondary necrotic cells with peripheral condensation of chromatin and ruptured cell membranes (yellow arrow) as well as apoptotic bodies (Red arrows) were also observed. **g** Photomicrograph post 2 h exposure to ELF-EMF in combination to AgNPs showing necrotic cells with mixed euchromatin and heterochromatin (Red arrows), ruptured cell membranes (Green arrows), intranuclear eosinophilic structures (Yellow  arrows) and shrunken apoptotic cells with irregular cell and nuclear membranes (Black arrows). **d&h** Control untreated MCF-7 cells examined at 1 and 2 h, respectively showing regular cells with hyperchromatic nuclei (Original magnification 100X, Oil)

Microscopic examination of MCF-7 cells treated with sole ELF-EMF for 1 h revealed the detection of swollen cells, swollen nuclei with mixed euchromatin and heterochromatin, ruptured cell membranes as well as intranuclear eosinophilic structures (Fig. 4a). Extending the exposure time to 2 h showed characteristic features of apoptosis such as shrinkage of cells and peripheral condensation of chromatin. Necrotic swollen cells with mixed euchromatin and heterochromatin and ruptured cell membrane were also observed (Fig. 4e). Apoptotic shrunken cells with shrunken nuclei, peripheral condensation of chromatin and irregular cell membranes were detected post 1 and 2 h treatment with AgNPs, respectively (Fig. 4b, f). Treatment with ELF-EMF in combination to AgNPs for 1 h revealed the presence of swollen necrotic cells and swollen nuclei with mixed euchromatin and heterochromatin and ruptured cell membranes. Shrunken apoptotic cells with peripheral condensation of chromatin in addition to secondary necrotic cells with peripheral condensation of chromatin and ruptured cell membranes as well as apoptotic bodies were also observed (Fig. 4c). Necrotic cells with mixed euchromatin and heterochromatin, ruptured cell membranes, intranuclear eosinophilic structures as well as shrunken apoptotic cells with irregular cellular and nuclear membranes were detected post 2 h exposure to ELF-EMF in combination to AgNPs (Fig. 4g). On the contrary, untreated cells showed regular appearance with hyperchromatic nuclei, respectively (Fig. 4d, h).

### Apoptosis

**Fig. 5:**
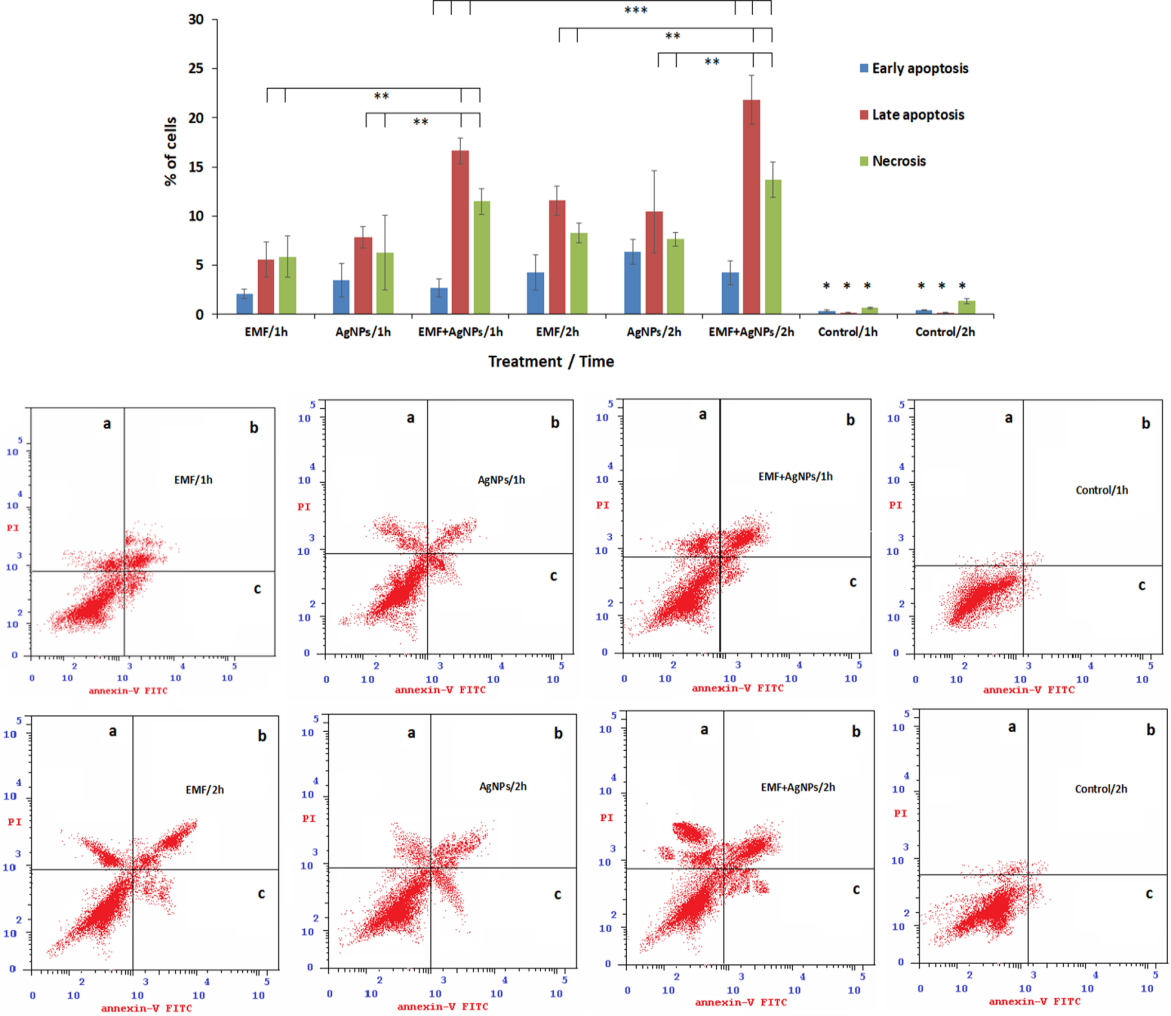
Evaluation of early and late apoptosis as well as necrosis post MCF-7 cells post exposure to ELF-EMF, AgNPs and ELF-EMF/AgNPs for 1 and 2 h interval using annexin V-FITC apoptosis detection kit. *P < 0.001, **P < 0.01, ***P < 0.05. **a** necrotic cells, **b** late apoptosis, **c** early apoptosis

Recorded data showed that the sole exposure to ELF-EMF and AgNPs or in combination significantly (P < 0.001) induced time dependent apoptosis compared to untreated cells. Also, a statistically significant (P < 0.01) induction of apoptosis post 1 h exposure to ELF-EMF-AgNPs combined form recording 16.65% ± 1.3 and 11.48% ± 1.3 of the analyzed cells in late apoptosis and necrosis, respectively, compared to lower levels of the detected cells (5.61% ± 1.8 and 5.87% ± 2.1) post treatment with sole ELF-EMF or AgNPs (7.82% ± 1.1 and 6.29% ± 3.8), respectively. Further exposure to ELF-EMF combined with AgNPs for 2 h significantly (P < 0.01) resulted in a marked elevation of early apoptotic and necrotic cells in the order of 21.84% ± 2.5 and 13.71% ± 1.8 compared to that in case of treatment with sole ELF-EMF (11.59% ± 1.5 and 8.26% ± 1.0) and AgNPs (10.45% ± 4.2 and 7.65% ± 0.7), respectively (Fig. 5).

### Cell cycle analysis

**Fig. 6:**
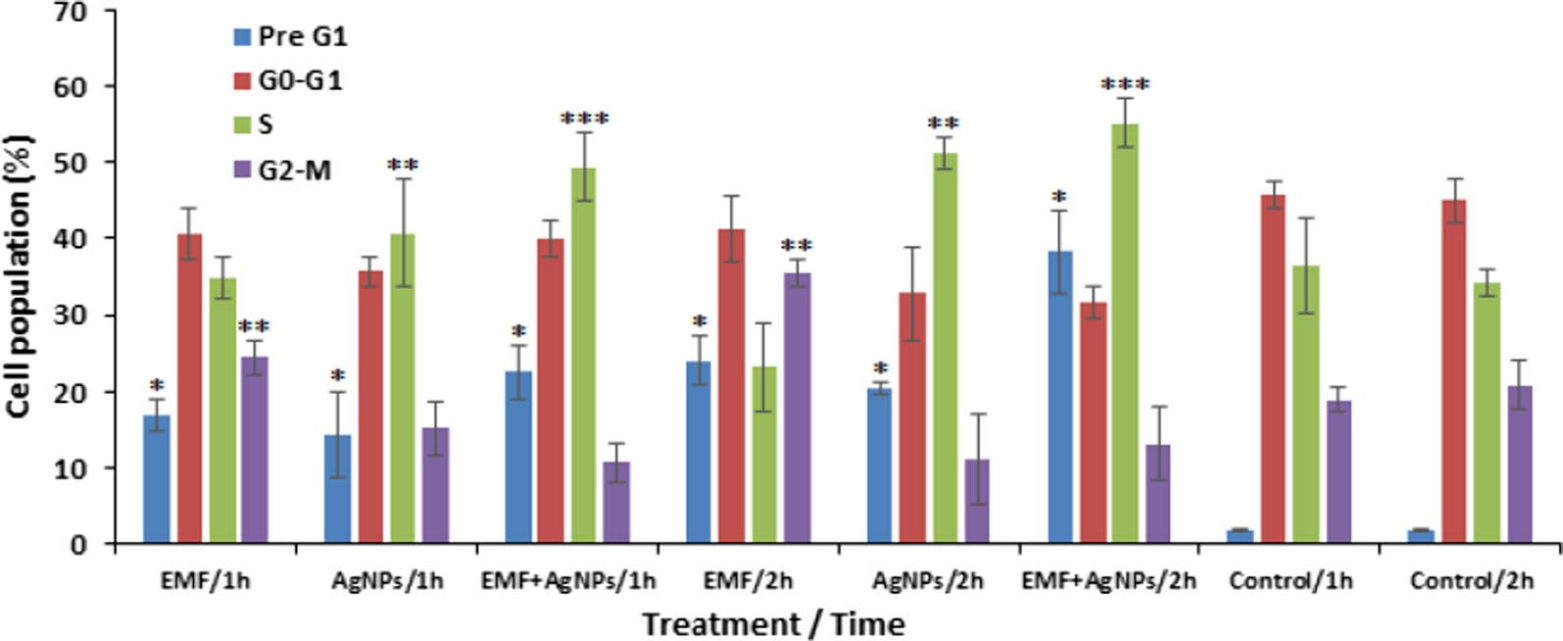
Evaluation of cell cycle profile post short-term exposure of MCF-7 cells to ELF-EMF and AgNPs as sole treatment as well as in combination at 1 and 2 h time interval using flow cytometry. *P < 0.001, **P < 0.01, ***P < 0.05

Recorded data revealed a time dependent apoptosis as indicated by the elevation of MCF-7 cells in Pre G1 phase (16.85% ± 2, 24.11% ± 3.1) post treatment with ELF-EMF for 1 and 2 h, respectively. Treatment with AgNPs resulted in a similar pattern of apoptosis induction recording 14.34% ± 5.5, 20.35% ± 0.8 of cells in Pre G1 phase post 1 and 2 h exposure, respectively. In the same context, MCF-7 cell treatment with ELF-EMF in combination to AgNPs induced a statistically significant (*p* < 0.001) time dependent higher levels of apoptotic cells (22.45% ± 3.5, 38.26% ± 5.4) post 1 and 2 h treatment, respectively. Also, sole treatment with ELF-EMF induced a significant (*p* < 0.01) time dependent G2/M phase arrest (24.52% ± 2.2, 35.53% ± 1.8) compared to untreated cells (18.95% ± 1.5, 20.76% ± 3.2). Whereas significant (*p* < 0.01) arrest during S phase (40.68% ± 7.1, 51.24% ± 2.2) was observed 1 and 2 h post exposure to sole AgNPs, respectively. Similarly, combined treatment with ELF-EMF and AgNPs resulted in a significant (*p* < 0.05) S phase arrest (49.43% ± 4.6, 55.17% ± 3.1) 1 and 2 h post treatment, respectively (Fig. 6).

### Gene expression profile

**Fig. 7:**
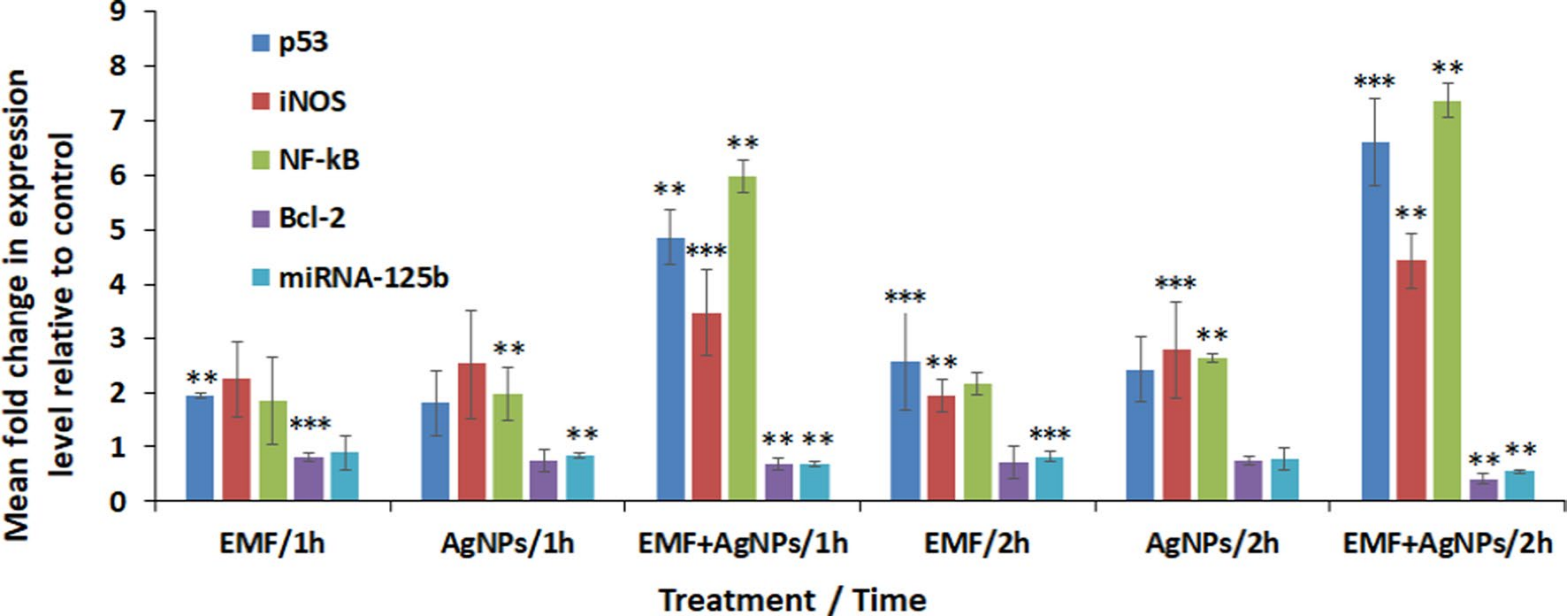
Evaluation of the expression profile of apoptosis related genes in MCF-7 cells post sole exposure to ELF-EMF and AgNPs as well as in combination at different time interval using real time PCR. **P < 0.01, ***P < 0.05

Expression level of apoptosis related genes was evaluated, it was found that p53, iNOS and NF-kB genes showed significant up-regulation recording 4.86-, 3.47- and 5.98-fold increase post 1 h exposure compared to 6.6-, 4.43- and 7.37-fold elevation post 2 h treatment, respectively. Down-regulation in the expression level of Bcl-2 and miRNA-125b in the order of 0.69- and 0.68-fold was observed post 1 h exposure to combined treatment compared to that recorded 2 h post treatment recording 0.42- and 0.55-fold change, respectively (Fig. 7).

### Biochemical analysis in MCF-7 cells

**Fig. 8:**
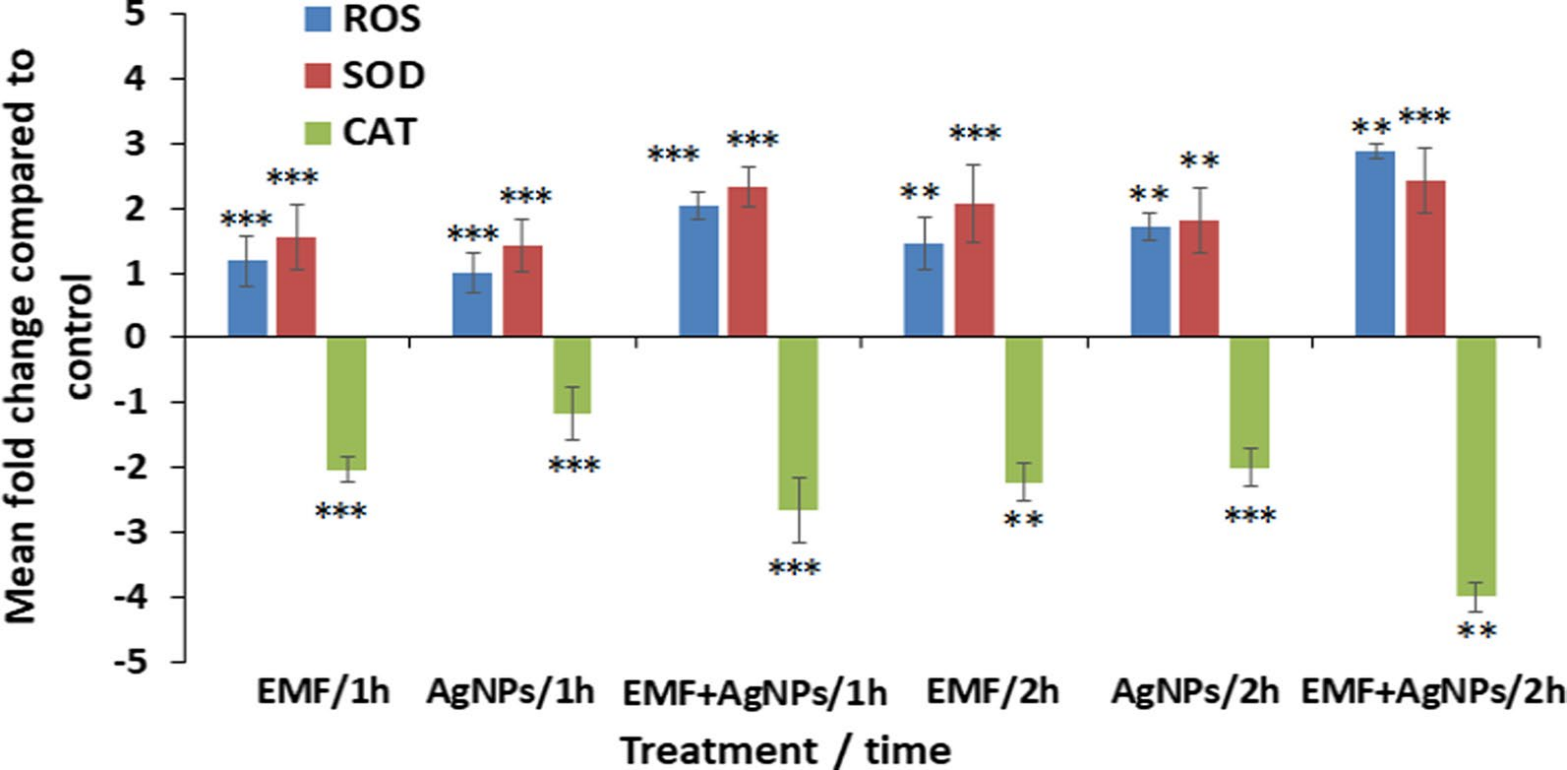
Assessment of the generation of ROS, SOD and CAT post MCF-7 cells exposure to ELF-EMF and AgNPs as sole treatment as well as in combination at 1 and 2 h time interval. **P < 0.01, ***P < 0.05

The oxidative stress induced 1 h post exposure of MCF-7 cells to ELF-EMF, AgNPs and ELF-EMF combined with AgNPs showed statistically significant (*P* < 0.05) elevated levels of the generated ROS recording 1.18- 1.0- and 2.04-fold compared to untreated cell control, respectively. Whereas, extending the exposure time to 2 h resulted in increased levels of ROS in the order of 1.45-, 1.72- and 2.88-fold compared to untreated cell control, respectively. Recorded data showed a significant (*P* < 0.05) elevated SOD levels recording 1.55-, 1.43- and 2.33-fold increase 1 h post treatment with ELF-EMF, AgNPs and ELF-EMF/AgNPs, respectively. While MCF-7 cells following 2 h treatment exhibited higher enhancement of SOD activity in the order of 2.06-, 1.8- and 2.43-fold compared to untreated cells, respectively. On the other side, the activity of catalase was significantly (*P* < 0.05) reduced post 1 h treatment with ELF-EMF, AgNPs and ELF-EMF/AgNPs by 2.04-, 1.17- and 2.66-fold, respectively. In the same context, the activity of catalase was also reduced post 2 h treatment by 2.23-, 2.0- and 4.0- fold, respectively (Fig. 8).

## Discussion

Multi-drug resistance is a threatening obstacle in the treatment of infectious diseases, where the misuse of broad-spectrum antibiotics has evoked antibiotic resistance among several human bacterial pathogens. AgNPs as well as ELF-EMF have attracted much attention in this field due to their recorded antibacterial potentials [6]. Thus, the present study tried to highlight the antibacterial potentials developed following exposure to these two weapons either alone or in combination. For this issue, *S. aureus* and *K. pneumonia* were selected as models for Gram-positive and Gram-negative bacteria, respectively. *S. aureus* is a commensal bacterium known to asymptomatically colonize the human skin, nasal passages, and gastrointestinal tract. *S. aureus* infections range from mild skin and soft tissue infections to more severe invasive diseases, such as endocarditis, bacteremia, sepsis, pneumonia, and osteomyelitis [31]. In the meantime, *K. pneumonia* accounts for a significant proportion of hospital-acquired urinary tract infections, pneumonia, septicemia as well as soft tissue infections and it has become multi-resistant to various types of antibiotics. *k. pneumonia* infections are also of clinical importance among patients in intensive care units with compromised immune systems such as cancer patients [6]. The present study aimed also to investigate the influence of short-term exposure to ELF-EMF and AgNPs either in sole or combined form at different time intervals. Thus, we selected 1 and 2 h time interval as a model for the short-term exposure time.

Similar to the current findings it was reported that AgNPs exhibited antibacterial activity against *E. coli* in a concentration and time dependent manner at a range of low concentrations in the order of 10 µM and 100 µM [32]. Many other studies demonstrated that the antibacterial potential of AgNPs isn’t only specific for Gram-negative bacteria (*E. coli*, *K. pneumonia and P. aeruginosa*), however its activity also extents to Gram positive (*S. aureus* and *B. subtilis*) bacterial strains. It was reported that the cytotoxic effect of AgNPs against bacteria may result from the oxidative dissolution of AgNPs and the release of Ag^+^ ions from AgNPs. The released Ag^+^ ions interact with sulfhydryl (-SH) groups of cell wall-bound enzymes and proteins, then interfere with the respiratory chain of bacteria resulting in disruption of the bacterial cell wall. In addition to the ability of the released ions to penetrate the bacterial cell wall and react with thiol groups of the proteins in cytoplasm as well as degrading the chromosomal DNA which subsequently result in failure of ATP production and chromosomal replication [33]. Another study examined *E. coli* post exposure to AgNPs using transmission electron microscope to observe the interaction between them. It was found that the positively charged Ag^+^ ions were attracted to the negatively charged bacterial lipopolysaccharides, induce the formation of holes in the bacterial cell wall and result in cell lysis [34]. It is important to note that the rate of dissolution of AgNPs to release Ag + ions is multifactorial, where it is dependent on several factors including their physicochemical properties such as the size, shape, concentration, capping agent and colloidal state of NPs as well as the presence of chlorine, thiols, sulfur, and oxygen [35].

In the current study, the recorded MICs and the viable count following treatment with AgNPs revealed that these particles showed greater inhibitory potentials towards Gram-negative bacteria compared to that observed in case of Gram-positive bacteria. Similar findings were reported in another study, where they demonstrated higher antibacterial activity of AgNPs against *E. coli* compared to that exerted on *S. aureus* [36]. That was attributed to the structural differences in the composition of the cell walls between Gram-positive and Gram-negative bacteria. Gram-negative bacteria have an outer layer of lipopolysaccharides and thin layer of peptidoglycan which confers for the overall lack of rigidity of the cell wall of Gram-negative bacteria. On the other side, Gram-positive bacteria have a three-dimensional rigid cell wall consisting of thicker layer of peptidoglycan which is formed of linear polysaccharide chains cross-linked by short peptides. The rigidity and cross-linking not only diminish the cell wall attachment sites for AgNPs in Gram-positive bacteria but also increase the difficulty of AgNPs penetration to the bacterial cell wall [19].

In the same context to the antibacterial potentials of AgNPs, other studies demonstrated the bacterial inhibitory activity of ELF-EMF. It was found that exposure of different Gram-positive and Gram-negative bacterial strains to ELF-EMF resulted in reduction in their growth rates with respect to control samples. In addition to the ultrastructural changes that were observed post treatment with this type of electromagnetic waves [37]. Also, another study reported a reduction in the percentage viability of *S. aureus* and *E. coli* post exposure to ELF-EMF. That was justified by the ability of these waves to alter the structure and function of the ion channels and efflux pumps of bacterial cell walls resulting in an alteration in the permeability of the bacterial cell wall to different molecules leading to cell death. Although many researchers tried to explore the effect of EMF on bacteria, but these effects are variable depending on the frequency and intensity of EMF, exposure time as well as the phase of bacterial growth, ingredients of the media, genetic properties, presence or absence of oxygen, and bacterial membrane features [38]. In accordance with the current findings, it was demonstrated that the antibacterial potentials of either AgNPs or EMF were highly effective against Gram-negative bacteria compared to that recorded in case of Gram-positive [2].

In agreement with the present study, it was reported that EMF is a versatile tool which could be successfully used for increasing the susceptibility of bacteria to antibacterial agents. They reported elevated antibacterial potentials when iron NPs were applied in combination to EMF against *Bacillus subtilis* and *E. coli* bacterial models. They attributed this elevated inactivation efficiency to the induction of higher local field gradients, hyperthermia, and motion of both the bacterial cells and magnetic NPs. Despite the ability of EMF to enhance the antibacterial potentials of different antimicrobials such as NPs, but their exact mechanism of action is still unclear and requires further investigations [39]. Thus, the present study evaluated the levels of ROS as well as the antioxidant enzymes (SOD and CAT) to explore the reason for the bacterial inhibitory potentials of AgNPs and ELF-EMF either alone or in combination. Current findings revealed that the antibacterial potential of AgNPs against *S. aureus* and *K. pneumonia* was directly proportional to the detected elevated levels of ROS in both bacterial models post treatment. Consequently, the recorded bacterial inhibitory potentials could be justified by the ability of AgNPs to induce oxidative stress in bacterial cells due to the production of ROS. It was reported that the positively charged silver ions released from AgNPs could induce oxidative stress in bacterial cells due to their interference with the normal function of the bacterial electron transport chain and thus facilitating the generation of ROS. ROS generation is primarily responsible for the bacterial death as it enhances lipid peroxidation but hindered ATP production and DNA replication [40]. At the same time, another study attributed the antibacterial potential of AgNPs to the combined effect of these particles on the bacterial components as previously demonstrated in addition to their ability to induce oxidative stress [41].

It is important to point out that, SOD and CAT are antioxidant enzymes which have been identified as critical modulators in AgNPs induced oxidative stress and are considered as bioindicators of increased ROS production. [42]. AgNPs induced oxidative stress in bacterial cells results in the generation of superoxide anion (O_2_^•−^). This free radical could be dismuted to hydrogen peroxide (H_2_O_2_) by the effect of SOD enzyme. The produced H_2_O_2_ is then quickly converted to H_2_O and O_2_ by CAT enzyme. The H_2_O_2_ produced by the effect of SOD could penetrate the bacterial membranes and interacts with ferrous ion (Fe^2+^) and thiol groups (-SH) of protein cysteines leading to inactivation of essential enzymes of the pathogen. Moreover, Fe^2+^ is oxidized during a Fenton reaction by H_2_O_2_ and generates hydroxyl radical, which in turn causes further destruction in the bacterial proteins, DNA, and lipids [43].

Similar to our results, a study found that the activity of SOD in AgNPs treated *P. aeruginosa* was elevated, however the activity of CAT was reduced post treatment. This study reported that AgNPs enhanced the activity of SOD enzyme resulting in an accumulation of H_2_O_2_. On the other hand, AgNPs treated cells failed to get rid of the accumulated H_2_O_2_ due to suppression of CAT activity by the effect of AgNPs. Accordingly, AgNPs treated bacterial cells showed elevated levels of cell death due to the enhancement of oxidative stress by the effect of the accumulated H_2_O_2_ [34]. That was suggested to be related to the formation of AgNPs-CAT complex which in turn resulted in conformational changes in CAT enzyme leading to an impairment of its enzymatic activity. In contrast, the formation of AgNPs-SOD complex has no influence on its enzymatic activity [44].

Regarding the anticancer potential of AgNPs and in agreement with the present findings, it was reported that AgNPs reduced MCF-7 cellular viability in a dose dependent manner recording an IC_50_ value of 6.28 μM [45]. It was reported that excessive production of ROS in cells by direct interaction with AgNPs and/ or dissolved silver ions is currently accepted as one of the main mechanisms of cellular toxicity of engineered nanoparticles in living organisms. Although ROS have many signaling and information functions, but it could also diminish the antioxidant defense system leading to damage of DNA, lipids and proteins [44]. That was obvious in the current study, where the recorded results revealed a time dependent reduction in CAT enzyme activity, which is considered an antioxidant enzyme, post treatment with AgNPs either alone or in combination to EMF. The observed CAT enzymatic activity reduction was also in parallel to apoptosis induction following treatment with AgNPs as well as production of ROS. This might be attributed to AgNPs induced generation of oxidative stress which was intensified by reduction of CAT enzymatic activity resulting in H_2_O_2_ accumulation as previously explained.

The recorded ability of EMF to induce apoptosis in MCF-7 cells could be related to some reports that  highlighted the ability of electromagnetic field to induce hyperthermia in tumor cells. It was demonstrated that hyperthermia could kill cancer cells but with limited hazards on healthy cells due to the well-known biophysical differences between the cancer cells and their healthy counterparts. Hyperthermia not only induce apoptosis in cancer cells but may also enhance the susceptibility of cancer cells to anticancer agents thus allowing the reduction of their administered doses [46]. That was obvious in the present findings, where the current results reported a time dependent ability of the combination between EMF and AgNPs to enhance apoptosis in AgNPs treated MCF-7 cells compared to the lower apoptosis induced in case of sole application of AgNPs.

In accordance with the current findings, a study investigated the biochemical consequences following exposure to EMF and suggested that the genotoxic events associated with exposure to EMF might be due to its ability to elevate the levels of free radicals which in turn led to DNA damage [47]. Another in vivo study demonstrated alteration in different oxidative stress biomarkers (SOD and CAT) as well as increased ROS levels in the brain of male rats following exposure to ELF-EMF for 2 h. It was also found that the exposure of murine squamous cell carcinoma line (AT478) to ELF-EMF for 16 min resulted in an increased ROS formation and SOD activities. Similar observations showed elevated ROS formation and induced cell death post 1 h exposure of breast cancer (MDA-MB-231) cells to EMF [48].

The ability of AgNPs and EMF to induce apoptosis either alone or in combination was proved in the present study using several techniques. For example, hematoxylin and eosin staining indicated the enhancement of apoptosis in MCF-7 cells. These results were confirmed via flowcytometric analysis using annexin V-FITC and PI staining. Also, a significant time dependent elevated late apoptosis was detected  following cellular treatment with EMF-AgNPs combination compared to that  in case of sole application of EMF and AgNPs as well as to untreated cell control.

Cell cycle analysis in addition to  the assessment of the gene expression profile of 5 apoptosis related genes were also performed to explore the correlation between the detected apoptosis and the influence of the applied treatments on cell cycle phases as well as the gene expression pattern. Recorded results proved the potential of EMF, AgNPs and  their combination to induce apoptosis as they significantly induced elevated level of cells in Pre G1 phase. The exposure to AgNPs was also accompanied by S phase arrest. On the other hand, following the exposure to EMF there was a significant elevation in the percentage of cells in G2/M phase. In agreement with the current findings, it was reported that treatment of MCF-7 cells with an IC_50_ concentration of AgNPs generated progressive accumulation of cells in the S phase of the cell cycle [45]. Another study reported the accumulation of MCF-7 cells in the G2/M phase 6, 12 and 24 h following exposure to EMF [49].

Regarding investigating the mechanism of action of the EMF and AgNPs on apoptosis at the molecular level. The expression levels of two pro-apoptotic genes (p53 and iNOS) as well as two anti-apoptotic genes (Bcl-2 and miRNA-125b) in addition to the impact on the expression level of NF-kB were evaluated following exposure to different treatments. In agreement with the current findings, a recent study reported that the expression levels of pro-apoptotic genes (p53, Bax and caspase-3) were significantly up-regulated, whereas the expression of the anti-apoptotic gene Bcl-2 was significantly down-regulated in AgNPs treated MCF-7 and colon cancer (HCT-116) cells [50].

The current study aimed to explore other novel mechanisms that might be involved in AgNPs or EMF induced cytotoxicity on MCF-7 cells. Thus, the expression profile of iNOS gene was investigated. Similar reports revealed that AgNPs elevated the expression level of iNOS genes as well as nitric oxide (NO) derived reactive species in human osteoblast cells [51]. However, it is essential to prove the generation of NO in our study on the transitional level not only on the molecular level. In accordance with the current findings, a study demonstrated down-regulation in the expression levels of various miRNA genes following treatment of cancer liver (HepG2) cells with different types of nanoparticles. The reported changes were highly induced by AgNPs followed by gold and iron nanoparticles and at  the same time they were accompanied by inhibition of both cellular proliferation and tumorigenesis [52].

It is worth to point out that NF-kB gene plays a dual role in apoptosis, where it could function as both a pro-apoptotic and anti-apoptotic regulatory factor within a single cell type [53]. It was also reported that the pro-apoptotic or anti-apoptotic function of NF-kB is determined by the nature of the apoptotic stimulus. Thus, the nature of the signals evoked by the respective death enhancers determines whether NF-kB induction leads to apoptosis or survival, suggesting that the modulation of NF-kB activity may present a new approach in cancer adjuvanted therapy [54]. A study demonstrated that the NF-kB pathway has been proposed to be a key factor contributing to the unusual phenotype and aggressiveness of breast cancer. In agreement with our results, it was reported that some up-regulated NF-kB-related genes could serve as novel therapeutic targets in breast cancer [55].

To the best of our knowledge, this is the first study that tried to explore the apoptosis induction potentials of the combination between ELF-EMF and AgNPs. Also, the mechanisms that may be involved in the antibacterial and anticancer potentials of the short-term exposure to ELF-EMF in combination to AgNPs have not been previously investigated. Current findings strongly suggest that the ability of ELF-EMF in combination to AgNPs to induce oxidative stress in bacterial and cancer cells via generation of ROS, SOD induction and catalase reduction could be responsible for their antibacterial and anticancer potentials. The significance of this study isn’t only related to exploring the antibacterial and the anticancer potentials of AgNPs and ELF-EMF either alone or in combination as new therapeutic approaches, but it spotlight on the effectiveness of the combination between these agents as an essential life-saving approach if this type of treatments could be applied clinically. That might offer greater health improvement especially in the immunocompromised cancer patients who are more vulnerable to develop infections with antibiotic resistant pathogens.

## Study limitations

This research study was performed on only two kinds of pathogenic bacterial models, one cancer cell model and only at one tested intensity (1 m Tesla) of the EMF. Further studies on different bacterial and cancer cell models as well as different exposure conditions are recommended.

## Conclusions

AgNPs and ELF-EMF could be considered as potential antibacterial and anticancer agents. The activities of these agents were enhanced upon their combinations in a time dependent manner even though at short exposure time. The recorded reduction in the bacterial viable count following exposure to these agents was higher against Gram-negative bacteria as compared to Gram-positive bacterial model. These antibacterial potentials were suggested to be related to the capability of these agents to induce oxidative stress by the generation of ROS. However, their effect was magnified via enhancing the antioxidant activity of SOD and on the other side reducing the activity of catalase enzyme resulting in elevated toxicity that might be attributed to H_2_O_2_ accumulation. Consequently, the combination between the tested agents could present a novel strategy for infection control and to overcome bacterial resistance. In the meantime, a time dependent induction of apoptosis was observed following treatment of MCF-7 cells with AgNPs, ELF-EMF as well as in combination. That was proposed to the ability of the tested treatments to significantly elevated the Pre G1 apoptotic phase of MCF-7 cells. Moreover, the exposure to AgNPs induced S phase arrest, whereas the EMF treatment was accompanied by accumulation of cells in the G2/M phase. Additionally, up-regulation in the expression level of p53, iNOS and NF-kB genes was observed, however down-regulation of the anti-apoptotic genes, namely Bcl-2 and miRNA-125b was detected post treatment. Biochemical analysis also shed light on the ability of both EMF and AgNPs to induce apoptosis via generation of oxidative stress. Finally, it could be concluded that AgNPs and ELF-EMF either in sole application or in combination could be considered as potential oxidative stress generating agents that might pave the way to solve the problem of antibiotic resistance especially in immunocompromised cancer patients and could successfully direct cancer cells to death.

## Data Availability

All data generated or analyzed during this study are included in this published article.

## Abbreviations

AgNPs: Silver nanoparticles
Bcl-2: B cell lymphoma-2
CAT: Catalase
ELF-EMF: Extremely low frequency electromagnetic field
FITC: Fluorescein isothiocyanate
iNOS: Inducible nitric oxide synthase
miR-125b: MicroRNA-125b
NF-kB: Nuclear factor-kappaB
PI: Propidium iodide
ROS: Reactive oxygen species
SOD: Superoxide dismutase

## References

[1] Malik B, Bhattacharyya S (2019). Antibiotic drug-resistance as a complex system driven by socio-economic growth and antibiotic misuse. Sci Rep.

[2] El-Kaliuoby MI, Khalil AM, El-Khatib AM, Shehata N (2021). Antibacterial synergism of electrospun nanofiber mats functioned with silver nanoparticles and pulsed electromagnetic waves. Polymers.

[3] Chan K, Morris GJ (2006). Chemoprevention of breast cancer for women at high risk. Semin Oncol.

[4] Johnston SR (1997). Acquired tamoxifen resistance in human breast cancer–potential mechanisms and clinical implications. Anticancer Drugs.

[5] Brown K (2002). Breast cancer chemoprevention: risk-benefit effects of the antioestrogen tamoxifen. Expert Opin Drug Saf.

[6] El-kaliuoby MI, Khalil AM, El-Khatib AM, Shalaby TI (2018). Synergistic antibacterial effect of silver nanoparticles and extremely low-frequency pulsed magnetic fields on klebsiella pneumoniae. Communications.

[7] Segatore B, Setacci D, Bennato F, Cardigno R, Amicosante G, Iorio R (2012). Evaluations of the effects of extremely low-frequency electromagnetic fields on growth and antibiotic susceptibility of escherichia coli and pseudomonas aeruginosa. Int J Microbiol.

[8] Falone S, Santini S, Cordone V, Di Emidio G, Tatone C, Cacchio M, Amicarelli F (2018). Extremely low-frequency magnetic fields and redox-responsive pathways linked to cancer drug resistance: insights from co-exposure-based in vitro studies. Front Public Health.

[9] Tofani S (2002). Electromagnetic field exposure system for the study of possible anti-cancer activity. IEEE Trans Electromagn Compat.

[10] Oh IR, Raymundo B, Jung SA, Kim HJ, Park JK, Kim CW (2020). Extremely low-frequency electromagnetic field altered PPARγ and CCL2 levels and suppressed CD44+/CD24− breast cancer cells characteristics. Bull Korean Chem Soc.

[11] Ashdown CP, Johns SC, Aminov E, Unanian M, Connacher W, Friend J, Fuster MM (2020). Pulsed low-frequency magnetic fields induce tumor membrane disruption and altered cell viability. Biophys J.

[12] Tofani S, Barone D, Berardelli M, Berno E, Cintorino M, Foglia L, Ossola P, Ronchetto F, Toso E, Eandi M (2003). Static and ELF magnetic fields enhance the in vivo anti-tumor efficacy of cis-platin against lewis lung carcinoma, but not of cyclophosphamide against B16 melanotic melanoma. Pharmacol Res.

[13] Akbarnejad Z, Eskandary H, Dini L, Vergallo C, Nematollahi-Mahani SN, Farsinejad A, Abadi MF, Ahmadi M (2017). Cytotoxicity of temozolomide on human glioblastoma cells is enhanced by the concomitant exposure to an extremely low-frequency electromagnetic field (100 Hz, 100 G). Biomed Pharmacother.

[14] Sriram MI, Kanth SB, Kalishwaralal K, Gurunathan S (2010). Antitumor activity of silver nanoparticles in Dalton's lymphoma ascites tumor model. Int J Nanomedicine.

[15] Banerjee I, Pangule RC, Kane RS (2011). Antifouling coatings: recent developments in the design of surfaces that prevent fouling by proteins, bacteria, and marine organisms. Adv Mater.

[16] Sanpui P, Murugadoss A, Prasad PD, Ghosh SS, Chattopadhyay A (2008). The antibacterial properties of a novel chitosan–Ag-nanoparticle composite. Int J Food Microbiol.

[17] Gomathi AC, Rajarathinam SX, Sadiq AM, Rajeshkumar S. Anticancer activity of silver nanoparticles synthesized using aqueous fruit shell extract of Tamarindus indica on MCF-7 human breast cancer cell line. J Drug Deliv Sci Technol. 2020;55:101376.

[18] Gurunathan S, Lee KJ, Kalishwaralal K, Sheikpranbabu S, Vaidyanathan R, Eom SH (2009). Antiangiogenic properties of silver nanoparticles. Biomaterials.

[19] Franci G, Falanga A, Galdiero S, Palomba L, Rai M, Morelli G, Galdiero M (2015). Silver nanoparticles as potential antibacterial agents. Molecules.

[20] AshaRani PV, Low Kah Mun G, Hande MP, Valiyaveettil S. Cytotoxicity and genotoxicity of silver nanoparticles in human cells. ACS Nano. 2009;3(2):279–90. 10.1021/nn800596w 19236062

[21] Franco-Molina MA, Mendoza-Gamboa E, Sierra-Rivera CA, Gómez-Flores RA, Zapata-Benavides P, Castillo-Tello P, Alcocer-González JM, Miranda-Hernández DF, Tamez-Guerra RS, Rodríguez-Padilla C (2010). Antitumor activity of colloidal silver on MCF-7 human breast cancer cells. J Exp Clin Cancer Res.

[22] Hsin YH, Chen CF, Huang S, Shih TS, Lai PS, Chueh PJ (2008). The apoptotic effect of nanosilver is mediated by a ROS-and JNK-dependent mechanism involving the mitochondrial pathway in NIH3T3 cells. Toxicol Lett.

[23] Raja G, Cao S, Kim DH, Kim TJ. Mechanoregulation of titanium dioxide nanoparticles in cancer therapy. Mater Sci Eng C. 2020;107:110303. 10.1016/j.msec.2019.110303 PMC7758999 31761191

[24] Raja G, Selvaraj V, Suk M, Suk KT, Kim TJ (2021). Metabolic phenotyping analysis of graphene oxide nanosheets exposures in breast cancer cells: metabolomics profiling techniques. Process Biochem.

[25] Raja G, Jung Y, Jung SH, Kim TJ. 1H-NMR-based metabolomics for cancer targeting and metabolic engineering–a review. Process Biochem. 2020.

[26] Raja G, Jang YK, Suh JS, Kim HS, Ahn SH, Kim TJ (2020). Microcellular environmental regulation of silver nanoparticles in cancer therapy: a critical review. Cancers.

[27] Alkawareek MY, Bahlool A, Abulateefeh SR, Alkilany AM (2019). Synergistic antibacterial activity of silver nanoparticles and hydrogen peroxide. PLoS ONE.

[28] Wayne PA, Clinical and laboratory standards institute. Performance standards for antimicrobial susceptibility testing. twenty-first informational supplement. M100–S21. CLSI. 2011: 100–121.

[29] Vijayakumar S, Ganesan S. In vitro cytotoxicity assay on gold nanoparticles with different stabilizing agents. J Nanomater. 2012. 10.1155/2012/734398

[30] Sholqamy MI, Abd-ElHamid ES, Mostafa AH, Mohamed AF, El-Said WA (2019). Monitoring the anticancer effects of two different gold nanostructures shapes towards Hep-2 Cells. Int J Med Nano Res.

[31] Jenkins A, Diep BA, Mai TT, Vo NH, Warrener P, Suzich J, Stover CK, Sellman BR (2015). Differential expression and roles of Staphylococcus aureus virulence determinants during colonization and disease. MBio.

[32] Orlov IA, Sankova TP, Babich PS, Sosnin IM, Ilyechova EY, Kirilenko DA, Brunkov PN, Ataev GL, Romanov AE, Puchkova LV. New silver nanoparticles induce apoptosis-like process in E. coli and interfere with mammalian copper metabolism. Int J Nanomed. 2016;11:6561. 10.2147/IJN.S117745 PMC5170787 28008247

[33] Liao C, Li Y, Tjong SC (2019). Bactericidal and cytotoxic properties of silver nanoparticles. Int J Mol Sci.

[34] Liao S, Zhang Y, Pan X, Zhu F, Jiang C, Liu Q, Cheng Z, Dai G, Wu G, Wang L, Chen L (2019). Antibacterial activity and mechanism of silver nanoparticles against multidrug-resistant Pseudomonas aeruginosa. Int J Nanomed.

[35] Ferdous Z, Nemmar A (2020). Health impact of silver nanoparticles: a review of the biodistribution and toxicity following various routes of exposure. Int J Mol Sci.

[36] Schiffman JD, Wang Y, Giannelis EP, Elimelech M (2011). Biocidal activity of plasma modified electrospun polysulfone mats functionalized with polyethyleneimine-capped silver nanoparticles. Langmuir.

[37] Inhan-Garip A, Aksu B, Akan Z, Akakin D, Ozaydin AN, San T (2011). Effect of extremely low frequency electromagnetic fields on growth rate and morphology of bacteria. Int J Radiat Biol.

[38] Amani S, Taheri M, Movahedi MM, Mohebi M, Nouri F, Mehdizadeh A. Evaluation of short-term exposure to 2.4 GHz radiofrequency radiation emitted from Wi-Fi routers on the antimicrobial susceptibility of Pseudomonas aeruginosa and Staphylococcus aureus. Galen Med J. 2020;9:1580. 10.31661/gmj.v9i0.1580 PMC8344163 34466555

[39] Novickij V, Stanevičienė R, Vepštaitė-Monstavičė I, Gruškienė R, Krivorotova T, Sereikaitė J, Novickij J, Servienė E (2018). Overcoming antimicrobial resistance in bacteria using bioactive magnetic nanoparticles and pulsed electromagnetic fields. Front Microbiol.

[40] Quinteros MA, Aristizábal VC, Dalmasso PR, Paraje MG, Páez PL (2016). Oxidative stress generation of silver nanoparticles in three bacterial genera and its relationship with the antimicrobial activity. Toxicol In Vitro.

[41] Pareek V, Gupta R, Panwar J (2018). Do physico-chemical properties of silver nanoparticles decide their interaction with biological media and bactericidal action? A review. Mater Sci Eng, C.

[42] Akter M, Sikder MT, Rahman MM, Ullah AA, Hossain KF, Banik S, Hosokawa T, Saito T, Kurasaki M (2018). A systematic review on silver nanoparticles-induced cytotoxicity: Physicochemical properties and perspectives. J Adv Res.

[43] Mourenza Á, Gil JA, Mateos LM, Letek M (2020). Oxidative stress-generating antimicrobials, a novel strategy to overcome antibacterial resistance. Antioxidants.

[44] Liu W, Worms I, Slaveykova VI (2020). Interaction of silver nanoparticles with antioxidant enzymes. Environ Sci Nano.

[45] Loutfy SA, Al-Ansary NA, Abdel-Ghani NT, Hamed AR, Mohamed MB, Craik JD, Eldin TA, Abdellah AM, Hussein Y, Hasanin MT, Elbehairi SE (2015). Anti-proliferative activities of metallic nanoparticles in an in vitro breast cancer model. Asian Pac J Cancer Prev.

[46] Hegyi G, Szigeti GP, Szász A. Hyperthermia versus oncothermia: cellular effects in complementary cancer therapy. Evid-Based Complement Altern Med. 2013;2013. 10.1155/2013/672873 PMC3638606 23662149

[47] Simkó M, Mattsson MO (2004). Extremely low frequency electromagnetic fields as effectors of cellular responses in vitro: possible immune cell activation. J Cell Biochem.

[48] Schuermann D, Mevissen M (2021). Manmade electromagnetic fields and oxidative stress—biological effects and consequences for health. Int J Mol Sci.

[49] Xu A, Wang Q, Lin T (2020). Low-frequency magnetic fields (LF-MFs) inhibit proliferation by triggering apoptosis and altering cell cycle distribution in breast cancer cells. Int J Mol Sci.

[50] Khan MS, Alomari A, Tabrez S, Hassan I, Wahab R, Bhat SA, Alafaleq NO, Altwaijry N, Shaik GM, Zaidi SK, Nouh W (2021). Anticancer potential of biogenic silver nanoparticles: a mechanistic study. Pharmaceutics.

[51] Zielinska E, Tukaj C, Radomski MW, Inkielewicz-Stepniak I (2016). Molecular mechanism of silver nanoparticles-induced human osteoblast cell death: protective effect of inducible nitric oxide synthase inhibitor. PLoS ONE.

[52] Brzóska K, Grądzka I, Kruszewski M (2019). Silver, gold, and iron oxide nanoparticles alter miRNA expression but do not affect DNA methylation in HepG2 cells. Materials.

[53] Lin B, Williams-Skipp C, Tao Y, Schleicher MS, Cano LL, Duke RC, Scheinman RI (1999). NF-κB functions as both a proapoptotic and antiapoptotic regulatory factor within a single cell type. Cell Death Differ.

[54] Kaltschmidt B, Kaltschmidt C, Hofmann TG, Hehner SP, Dröge W, Schmitz ML (2000). The pro-or anti-apoptotic function of NF-κB is determined by the nature of the apoptotic stimulus. Eur J Biochem.

[55] Lerebours F, Vacher S, Andrieu C, Espie M, Marty M, Lidereau R, Bieche I (2008). NF-kappa B genes have a major role in inflammatory breast cancer. BMC Cancer.

